# Effect of Flexible Sacrum Position on Maternal and Neonatal Outcomes in Public Health Facilities, Amhara Regional State, Ethiopia: A Quasi-Experimental Study

**DOI:** 10.3390/ijerph19159637

**Published:** 2022-08-05

**Authors:** Marta Berta Badi, Solomon Mekonnen Abebe, Mulat Adefris Weldetsadic, Kyllike Christensson, Helena Lindgren

**Affiliations:** 1 Department of Women’s and Family Health, School of Midwifery, College of Medicine and Health Sciences, University of Gondar, Gondar 6200, Ethiopia; 2 Institute of Public Health, College of Medicine and Health Sciences, University of Gondar, Gondar 6200, Ethiopia; 3 Department of Gynecology and Obstetrics, School of Medicine, College of Medicine and Health Sciences, University of Gondar, Gondar 6200, Ethiopia; 4 Department of Women’s and Children’s Health, Karolinska Institute, 17164 Stockholm, Sweden

**Keywords:** Ethiopia, flexible sacrum position, supine position, maternal outcome, neonatal outcome

## Abstract

Restricting women giving birth in health care facilities from choosing the most comfortable position during labor and birth is a global problem. This study was aimed to examine the effect of flexible sacrum birth positions on maternal and neonatal outcomes in public health facilities in Ethiopia’s Amhara Region. A non-equivalent control group post-test-only design was employed at public health facilities from August to November 2019. A total of 1048 participants were enrolled and assigned to intervention or control groups based on their choice of birth position. Participants who preferred the flexible sacrum birth position received the intervention, while participants who preferred the supine birth position were placed in the control group. Data were collected using observational follow-up from admission to immediate postpartum period. Log binomial logistic regression considering as treated analysis was used. Of the total participants, 970 women gave birth vaginally, of whom 378 were from the intervention group, and 592 were from the control group. The intervention decreased the chance of perineal tear and poor Apgar score by 43 and 39%, respectively. The flexible sacrum position reduced the duration of the second stage of labor by a mean difference of 26 min. Maternal and newborn outcomes were better in the flexible sacrum position.

## 1. Introduction

Childbirth is one of the most significant events in a woman’s life. Therefore, childbirth practices are essential to the woman’s and her child’s health and well-being [1]. The term childbirth positions, or maternal birthing positions refers to the physical postures adopted by a pregnant woman during the labor and birthing process. Birth positions are frequently described as upright, lateral, or supine (SP) [2]. Some upright or lateral positions can be characterized as flexible sacrum positions (FSPs), by which weight is taken off the sacrum, thereby allowing the pelvic outlet to expand and allow flexibility in the sacroiliac joints. The FSPs were kneeling, standing, squatting, all-fours, lateral position, and giving birth on the birth seat [3,4,5]. Given all these possibilities, it is essential to provide the women with information about results from studies attempting to determine differences in comfort level associated with differences in birth positions. Then in maternal care settings where women are allowed and/or encouraged to express their preferences, the women can make informed choices [6]. Women’s birth positions have changed dramatically, in high-income countries since the inception of obstetrics in the 17th century. Until changes were made, the most adopted positions were upright positions [7,8,9,10].

Medicalization of childbirth came into practice following the application of the forceps, continuous fetal heart rate monitoring, and analgesia to relieve labor pain, and leading to the supine position becoming the norm in high-income countries. A review of maternal birthing positions found that medicalization of childbirth was accompanied by making birth attendants responsible for choosing birth positions that they found suitable [11]. Generally, the women themselves were not consulted [8,10].

In another literature review, most women in high-, mid-, and low-income countries give birth in the SP in health care facilities [12,13,14,15,16]. Women choose to give birth at home since they are asked to adopt an unfamiliar birthing position in health facilities. So, women will choose a variety of movements and positions to cope with labor pain if they are left to their own decision. Women instinctively use standing, walking, rhythmic swaying movements, and positions in response to pain or other sensations during labor (1). It is well known that throughout the ages and across human cultures, women giving birth alone have preferred to do this with their bodies vertical in sitting or squatting positions by grasping a tree, ropes or knotted piece of cloth and generally avoiding lying flat on their back [14,17,18,19,20].

Studies found that where the birthing process included spontaneous pushing and letting the woman choose her position for birth, there was a reduction in the occurrence of perineal injuries [5,21,22]. Evidence from one randomized trial suggests that upright positions during the second stage of labor provide several benefits: a lower risk of abnormal fetal heart rate patterns, less pain, less use of vacuum/forceps and less episiotomy/perineal tear. The study indicated that the upright position also shortens the second stage of labor and reduces augmentation [23]. Furthermore, studies showed that FSP reduces the incidence of operative vaginal delivery, instrumental delivery, caesarean section, episiotomy, severe perineal trauma and vaginal tear, severe pain, and shortens the duration of the first stage of labor and active pushing phase in the second stage of labor. However, other studies showed that FSP increase the incidence of first- and second-degree perineal trauma and estimated blood loss greater than 500 mL [14,24,25,26,27,28,29,30], and no difference in prevalence of Neonatal Intensive Care Unit(NICU) admissions [28,29,31]. Perineal laceration(s) can result in serious maternal morbidity and even mortality in low and middle-income countries, including Ethiopia. Perineal tear is highly prevalent in Ethiopia: one study showed prevalence of perineal tear was 38.4% in Atat hospital [32].

The World Health Organization(WHO) and the Respectful Maternity Care Charter recommend that women should be allowed to use labor and birthing positions they prefer and be involved in the decision [33,34]. Additionally, Ethiopia’s Ministry of health recommended that giving more attention to the choice of birthing position as a component of respectful maternity care is a key to good care for women who do not have access to health facilities, and who do not give birth in such facilities. Whether this approach is fully applicable has not yet been determined [35]. Despite these findings and recommendations, 89% of laboring women in Ethiopia gave birth in supine positions [13,36], and only about two in five women in health centers and one in five women in hospitals were given choices for birth position [35].There were scarce interventional studies done to estimate the effect of FSP on maternal and neonatal outcomes in the study area, as well in Ethiopia. Therefore, we designed our study to take the first steps in investigating the effect of the FSP on maternal and neonatal outcomes in public health facilities of Amhara region in Ethiopia.

## 2. Materials and Methods

### 2.1. Study Design, Setting and Period

Non-equivalent control group post-test-only design was conducted in public health facilities of the Amhara region in Ethiopia, from August to November 2019. Ethiopia is located at the horn of Africa, and is bordered by Eritrea, Djibouti, Somalia, Kenya, South Sudan, and Sudan. It encompasses about 1.1 million square kilometers area of land and is characterized by varied topography and different climatic conditions. The estimated population is more than 107 million, the average life expectancy is 64.6 years, and the growth rate is 2.6% per year, making Ethiopia the second most-populous country in Africa. The present study was conducted in the Amhara region which is about 784 km from Addis Ababa. The study was conducted in public health facilities of four towns (Debark, Debretabor, Weldia and Finoteselam) found in Amhara region. Amhara region is the second most-populous region in Ethiopia with over 20 million people. There are 55 hospitals (5 referral hospitals, 2 zonal hospitals and 48 district hospitals). Four district hospitals and six health centers were selected for the study.

**Participants:** All pregnant women who came for labor and birth services with low-risk pregnancy to the selected public health facilities during the study period were included in the study population. The inclusion criteria were women in the active phase of first stage of labor with spontaneous onset, singleton fetus, live, cephalic presentation, and gestational age between 37 and 42 weeks. Pregnant women with obstetric and medical difficulties were excluded, as were women with communication problems or hearing difficulties.

#### Sample Size Determination

The sample size was estimated using Epi Info 7.2.1.0 software with the assumption of a 5% level of significance (2-sided) and power of 80%, experimental to the non-experimental ratio of 1:1 with the proportion of second-degree perineal tear 70.7% among the intervention group and 78.3% among the control group [37]. The minimal sample size needed was 1190 individuals, of whom 530 were in the intervention group (FSPs) and 660 were in the control group (SP), considering a non-response rate and loss to follow-up of 10%. 

A two-stage random sampling technique was utilized to select the study participants. First simple random sampling was used to select ten public health facilities. Second, a systematic random sampling technique was used to determine the study participants. The sample size was proportionally allocated based on the average number of laboring mothers attending for labor and birth at the selected health facilities in the previous three months; this was as determined from the monthly report from each facility. At the time of allocation women were allowed to choose position and based on that preference the selected mothers were assigned either to the intervention group (FSPs) or to the control group (SP).

**Intervention group:** comprised the study participants who preferred the flexible sacrum position (FSPs) and received the intervention. If the study participants chose either of the FSPs such as kneeling, standing, squatting, all-fours, lateral, or the birth seat positions, reported as FSPs.

**Control group:** comprised the study participants who preferred the supine position (SP) and received the routine care. If the laboring mother liked either of the SP such as lithotomy, semi-sitting, recumbent, or semi-recumbent positions, they were reported as SP (NFSPs).

First, there was information creation session (about different types of birth positions, so that they can make informed decision to choose between the FSP and supine). This was done for all mothers when they came for labor to the health facilities. The only chance given to the control group was to choose between the FSP or SP (supine position), but nothing was said about which group would be the intervention or the control. 

**Intervention package**: To apply the new birth position (FSP) in Ethiopia, the following activities were provided. Midwives were trained to attend to the participants who choose the flexible sacrum position (FSP).

1. The women in the FSP were allowed to change position among the FSP (standing, kneeling, squatting, all four, seat birth and lateral positions);
2. For laboring women to change among the above-listed FSP positions, they were allowed to have a companion with them to assist when standing, walking, squatting and on birth seat position.
3. Continuous follow-up and labor support by the trained midwives.
4. The trained midwives applied different pain relief methods and perineal support: different types of perineal support methods were applied in different FSP positions to reduce risk of perineal tear. All the above intervention strategies were applied to the intervention group only, because it needs by its nature to have safe labor and delivery services. To apply the intervention, equipments used were mattresses (for all four positions, kneeling, sometimes for squatting position), seats for birth seat position (for laboring mothers and the midwives attending labor in birth seat position), and screen sheets (to separate mothers in different types of birth position).

### 2.2. Outcome Ascertainment

The primary outcome variable in this study was a perineal tear, which is classified into four types: first-degree perineal tears with injury to perineal skin and/or vaginal mucosa; second-degree perineal tears with injury to the perineum involving perineal muscles but not involving the anal sphincter [38]; third-degree perineal tears with an injury to the perineum involving the anal sphincter complex; and fourth-degree perineal tears with an injury to the perineum involving the anal sphincter complex, external anal sphincter, and internal anal sphincter and anorectal mucosa [39].

Perineal tears were measured: if the study participants developed first, second, third or fourth-degree perineal tears that fulfill the above definition and need to be sutured and labial tear sutured, the perineal tear was recorded as **“Yes”**. If the study participants developed abrasion but not sutured and intact perineum, the perineal tear was recorded as **“No” [40]**. 

Secondary outcome variables: duration of the second stage of labor, instrumental delivery, mode of delivery, low Apgar score (Appearance, Pulse, Grimace, Activity and Respiration) score and sent to NICU were examined as a secondary variable. 

Duration of second stage: this was defined as the time from fully dilated cervix when peaked till the birth of the baby. A prolonged second stage of labor was recorded if first-time mothers (primigravida mothers) were in that stage for >3 h and for experienced mothers(multi gravid mothers > 2 h) [41]. 

**APGAR score**: was defined as the 5th minute APGAR score of <7 [42,43,44,45] and neonate sent to NICU for neonatal asphyxia. 

### 2.3. Data Quality Control, Data Collection Tools and Procedures 

Midwives at the study’s public health facilities were trained, with a total of 104 midwives receiving the professional training for two days of theory and three days of clinical practice (in the skills lab and clinical settings). The training was about the types of birth positions that can be used during labor and birth, their advantage and disadvantage, and how to assist and provide pain relief methods for participants who preferred the FSPs. Midwifery experts provided the training, which was needed because the midwives had no prior experience of attending women giving birth in the FSPs. The trained midwives working in maternal health care units in the study area could educate pregnant women at ANC and when they came for labor and attend birth in FSPs. After the training they continued to practice for one month with follow-up supervision per week before beginning to attend participants in the study and before the actual data collection. Two expert midwives did the follow-up supervision. After a practical follow-up supervision period, the data collection team of six first degree midwives and two MSc midwives (for supervision) were assigned to each hospital. Two first degree midwives and one MSc midwife were assigned for the data collection and supervision at each health center. The test instrument was validated content wise. Before the actual data collection began, the supervisors and data collectors received two days training on how to approach and interview laboring mothers, on the questionnaires, understanding the checklists, and when and how to record the outcomes at the time of observation. The pre-tested and semi-structured questionnaire was used by the data collectors and supervisors to collect the data, after they had been provided with data collection guidelines. The questionnaires have socio-economic, demographic and obstetric variables, and there were also checklists for the outcome variables.

The trained midwives informed the study participants about the types of various birth positions after collecting baseline socio-demographic and obstetric data from the chosen study participants, and they advised them to occupy whatever positions felt natural to them. The intervention and control groups were then created based on the women’s indicated preferences for birth positions. However, the study participants did not know which group would receive the intervention. Observational follow-up was maintained from admission until the immediate postnatal period, and information on the positions taken during the second stage of labor and the exact times of delivery was gathered. Additionally, the peak time when the cervix was fully dilated, the time when passive pushing started, the time of active pushing (expulsive pushing), and the actual time of birth were all recorded to measure the duration of the second stage of labor. Data were also collected on the perineal tears and other maternal and neonatal outcomes on direct observation, drawn from the maternity clinical charts, delivery charts and by asking the midwives who attended the labor and delivery. This helped the data collection team to maintain a consistent method for conducting the interviews, hence avoiding the possible introduction of bias during data collection [46].

### 2.4. Data Analysis

Data were analyzed according to required standards: data were cleaned, coded, and entered by Epi-info version-7 and stored in a Microsoft Access database file format, before being exported to STATA version-14 for analysis. Data management for all fields was conducted by looking at data ranges as well as looking for missing data. Descriptive analysis was done using frequencies and percentages. The chi-square test for categorical and independent t-test for continuous variables were employed to compare the intervention and control groups. The results were presented using texts, tables, and figures. Bi-variable log-binominal regression analysis was used to estimate the effect of FSP on the maternal and neonatal outcomes with relative risk at 95% confidence interval. A *p*–value of ≤ 0.05 was considered statistically significant. There was no need to fit multi-variable log-binominal regression analysis since the explanatory variables were uniformly distributed between the intervention and control groups.

## 3. Results

A total of 1190 laboring women were recruited for the study. Of these, 142 were excluded from the study (98 women developed complications, and 44 were entered in to the second stage of labor before the actual information gathering session and allocation to either group was completed). Thus, women enrolled for follow-up were 1048 of those; 970 women completed labor vaginally and 78 (43 from the intervention and 35 from the control) completed with caesarean section. 

**Figure 1:**
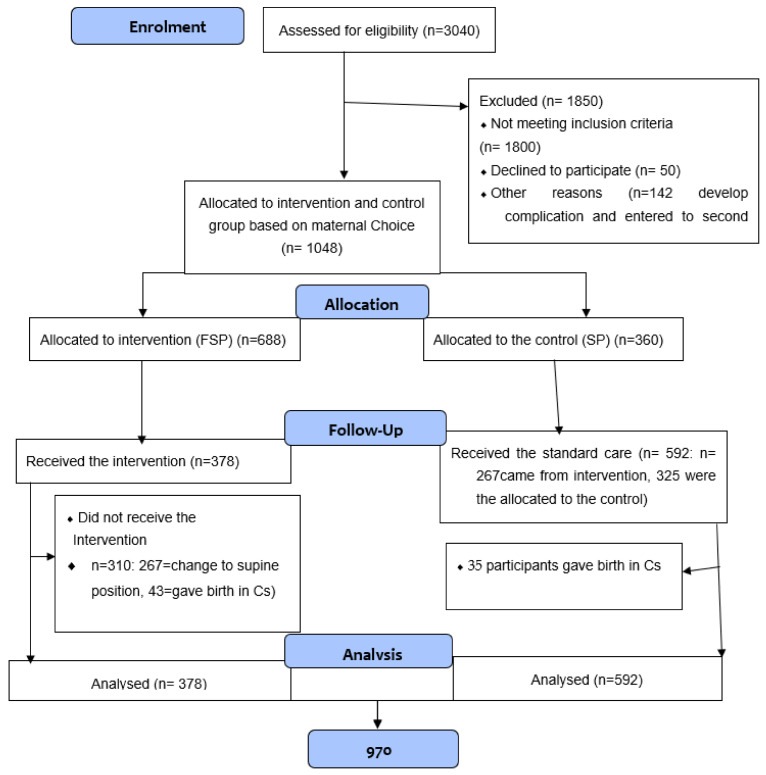
Flow diagram for participants’ recruitment, enrollment, follow-up, and analysis in the study in Amhara public Health facilities, 2019.

At baseline, a total 1048 participants, 688 (65.6%) mothers preferred the FSPs and 360 (35.5%) preferred the SP. At the time of birth from the total 970 participants, 378 (39%) participants gave birth in the FSPs and 592 (61%) gave birth in the SP; because the 267 (41.4%) participants changed their position from intervention to the control group during the follow-up period after choosing FSP at the allocation: Figure 1.

**CONSORT 2010 Flow Diagram (Quasi-experimental)**

### 3.1. Socio-Demographic Characteristics of the Study Participants

The mean (SD) age of the laboring women was 27 (5.66); the majority 730 (69.7%) were urban residents; 952 (90.8%) were orthodox by religion, and 995 (94.9%) were married (Table 1).

The obstetric characteristics of the study participants that compared between the groups, did not show statistically significant differences. Table 2.

### 3.2. Flexible Sacrum Birth Positions used at the Time of Birth 

**Figure 2:**
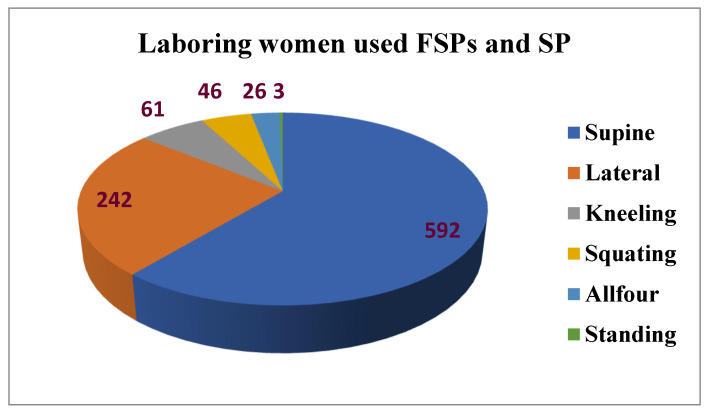
Number of the study participants who used the flexible sacrum positions and supine position, in Amhara public Health facilities, 2019.

Most laboring mothers used the supine position that is used by the control group 592 (61.12%), which is routinely used. The second most-used birth position was the lateral position 242 (25%), which is among the intervention positions (FSPs) and the remaining positions were also the FSPs shown as follows. Figure 2.

### 3.3. Maternal and Neonatal Outcomes in the Intervention Group Compared to the Control Group

**Figure 3:**
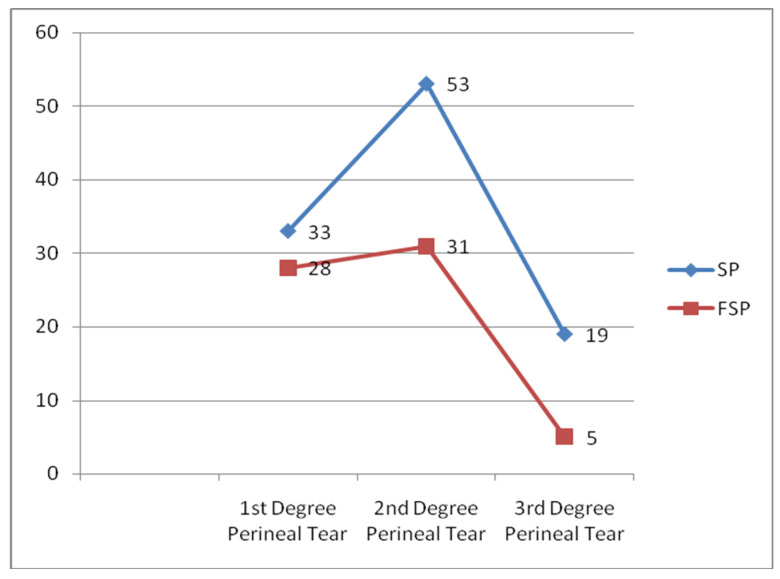
Number of the study participants with degrees of perineal tear compared between the intervention and the control group, in Amhara public Health facilities, 2019.

The overall proportion of perineal tears in this study was 17.4% (95%CI: 15.2, 19.4). A higher proportion of perineal tears was seen among the control group 124 or 20.9% (95%CI: 17.8, 24.4) than the intervention group 45 or 11.9% (95% CI: 9.0, 15.6). Of this, 53 (9.0%) of the participants in the control group experienced second-degree perineal tears, and 31 (8.1%) in the intervention group, while 19 (3.2%) of the participants in the control group experienced third-degree perineal tears and 5 (1.3%) in the intervention group (Figure 3).

**Figure 4:**
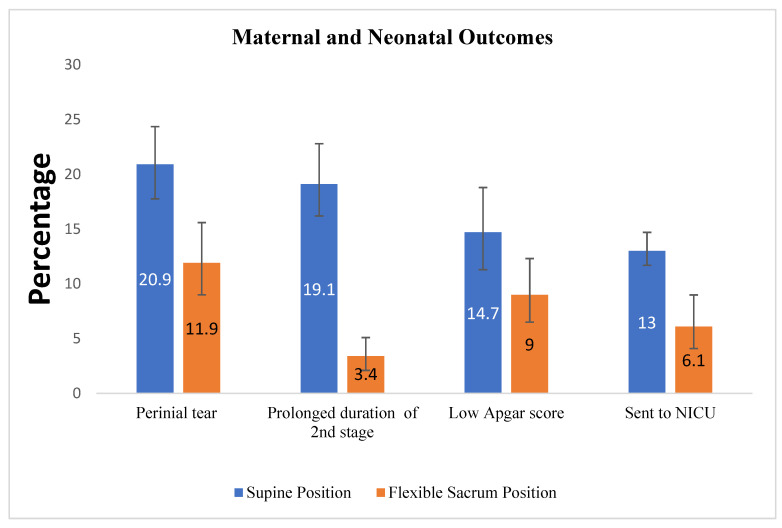
Percentage of maternal and neonatal outcomes compared in the intervention and control group in Amhara public Health facilities, 2019. The percentage was first calculated with 100%, for categories of outcomes, then the result was compared between the intervention and control groups. Perineal tear was for the “Yes” response.

The mean duration of the second stage of labor was shorter (56 min (sd ± 180)) among women in the intervention group than in the control group (82 min (±24)) by a mean difference of 26 min (*p*-value < 000, CI: 23–28 min). A lower proportion of prolonged duration of the second stage of labor has been seen among women in the intervention group 22 (4.1%) than in the control group 62 (15.6%), less proportion of instrumental delivery in the intervention 26 (6.88%) group than the control 64(10.81%); similarly, the mode of delivery the caesarean section is less in proportion among the intervention group 43 (6.3%) than the control group 35 (9.7%). Among the neonatal outcomes, having had low Apgar scores of <7 was lower in the intervention group 34 (9%) than in the control group 87 (14.7%). The proportion of neonates sent to NICU were lower among the intervention group 23 (6.1%) than in the control group 77 (13%) (Figure 4).

### 3.4. Effect of Flexible Sacrum Position on Maternal and Neonatal Outcomes

Bi-variable Log binominal regression analysis was used to estimate the effect of the intervention on perineal tear and low Apgar score. Risk of perineal tear was decreased by 43% (RR = 0.57 (0.41, 0.78)) and low APGAR score was decreased by 39% (RR = 0.61 (0.42, 0.89)) due to the intervention Table 3.

## 4. Discussion

Women who gave birth in the flexible sacrum position had a 43% reduced risk of perineal tear related to the birth. Perineal tear may result in subsequent problems with sitting, breast feeding and using the toilet [47]. Our findings were highly supported by results from a population-based study [48], that similarly allowed mothers in labor to choose among different flexible sacrum positions. The differences in birth positions were compared with one another in that study, where the lateral position was found to be highly protective from perineal tear. Another similar study was conducted by Edqvist et al. [37], which showed that FSP protects women from developing second-degree perineal tears. Edqvist et al. also highlighted that ignoring maternal choice of position would be a potential source of bias, while respecting their choice to involve them in the decision-making process may lead to better maternal outcomes. Long-term problems of women having perineal injuries are dyspareunia [49], lower levels of sexual arousal and orgasm [50], as well as pelvic organ prolapse later in life, and an influence on their quality of life [50,51].

The FSP was associated with a lower proportion of second- and third-degree perineal tears. Previous studies supported this result. The study done by Zhang showed 43.4% of women developed perineal tear among the supine group and 10.5% of women developed perineal tear among the FSP group [52]. The other study was by Edqvist et al. [37], which found that 78.3%of the supine group experienced second-degree perineal tear whereas 70.7% in the FSP group. The lower proportion of second- and third-degree perineal tears in the intervention group in the present study was not, however, in agreement with results from Gupta, Dabral and Shorten [31,40,53], who reported that the proportion of second- and third-degree perineal tears was greater in women who had used the FSP birth position. Possible reasons for the study by Dabral could be that mothers were not offered alternative, upright positions, and participants were offered only the kneeling position. There were recommendations from different previous studies to allow women to use the position they believed would be most comfortable, and even Dabral recommended to allow mothers in labor to choose from alternative birth positions [31,53]. In the case of Shorten’s study, there was a chance of changing to their comfortable position. However, in this study, some deliveries were attended by physicians and student midwives, who were not trained to attend deliveries in the new FSP. Thus, they had no prior experience in using alternative positions for vaginal deliveries. 

In contrast, in the present study, midwives attended deliveries with more than six months of work experience in labor and delivery service. They were also trained to attend labor and deliveries with women in a dynamic position. Mothers in the current study were assigned based on their free choice of comfortable birth positions, and this free choice is believed to give psychological satisfaction and result in a reduction in stress and pain. Free choice is also an essential component in quality maternal care [31,54]. Other studies did not show differences in the proportion of perineal tears using different positions during the second stage [17,48,55]. In the case of the newborns in the present study, the intervention group had a 39 % reduction in the risk of Apgar score <7 and fewer infants were referred to NICU. Free ambulation and being in comfortable positions decrease fetal distress and neonatal Asphyxia, and this, in turn, can result in a decrease in the incidence of low Apgar scores [56,57].

Our result is in line with a study by Thilagavathy [17]. Our findings are inconsistent with those from China, India, and Turkey [31,52,56], showing no difference for the neonates referred to NICU between the intervention and control groups. This could be due to the difference in the study settings such as in high-income countries like Turkey and China, where all necessary materials are available, with patients having private rooms being able to ambulate freely. In the present study, mothers were encouraged to choose among the FSP alternatives, but limited alternatives were given in the contrasting studies. FSP prevent compression of aortocaval blood vessels, which will decrease in the incidence of Asphyxia, which is the main reason for referral to NICU [50,56,58].

This is in line with results from a study by Dabral [31], which compared the kneeling and supine positions; kneeling is one of the flexible sacrum positions and was a factor in reducing referrals to NICU. Those results are, however, inconsistent with results reported by O. Moraloglu [56], which showed no difference for referrals to NICU. In the case of Moraloglu’s study, only nulliparous women participated, and nulliparity by itself is the main cause of a prolonged second stage of labor; in turn, the protracted second stage of labor will be the cause of Asphyxia [49].

The mean duration of the second stage of labor was shortened by a mean difference of 26 min for women who preferred FSP. The flexible sacrum position allows gravity to be a factor affecting uterine contractions and fetal alignment to the birth canal [59]. The current finding is supported by several studies [17,30,31,49,55,56], but it is inconsistent with the reports in the Cochrane Database of Systematic Reviews, or a primary study conducted by Gupta. JK et al., [50,53]. One possible reason for differences is if results from deliveries attended by untrained providers, and deliveries where the mothers were allowed only either supine or squatting positions, so it could be difficult to compare with results from studies conducted differently. In the Cochrane Database of Systematic Reviews case, 22 trials were reviewed, suggesting that mothers in labor should be allowed to use any upright or lateral position. Still, the review did not show a significant reduction in the duration of the second stage of labor. In addition, there is a time difference; the primary studies reviewed have had less duration difference except in the case of two studies.

The first strength of this study is that it was conducted in a multicenter setting involving both hospitals and health centers, which enabled us to include births attended by midwives in different environments. Secondly, all midwives working in the ANC and delivery units in all the study sites were trained and informed about all alternative birth positions and how to assist laboring mothers based on their choice of position. This made them counsel the mothers and explain the chance of choosing a birth position and encourage them to feel free to select the position. The data collectors and supervisors were trained and provided with data collection guidelines.

There were limitations in this study. The first limitation is that not all participants received information about birth positions during pregnancy and were only informed during labor. This may decrease the acceptance of the new position, because it does not give time for the mother to make the appropriate informed decision. The second limitation was that non-equivalent control group post-test-only design was used to assess the effect of flexible sacrum position. This design has some advantages as it does avoid the situations when the post-test results can be influenced by the results of a pretest. However, because there is no pretest, it may be more difficult to determine: the magnitude of the maternal or neonatal outcomes in the current study. The third limitation was the high risk of attrition bias with this study design. This is mainly due to pregnant participants quitting the study for different reasons between the study groups, making the groups unequal. That is why the risk of attrition bias does seem higher in this study. The absence of a pretest makes it very hard to detect and control this bias. Moreover, it could be more difficult to determine the magnitude of the maternal or neonatal outcomes in the current study.

## 5. Conclusions

Women who gave birth in an FSPs had significantly reduced risks of perineal tears. The neonates had reduced risks of a low Apgar score when their mothers had given birth in an FSP. The reduced perineal pain and the presence of good and vital signs in the infants might lead to early interaction between the mother and her baby as well as facilitates the initiation of early breastfeeding.

## 6. Clinical Implication and Recommendation

Women should be able to choose the birth position they find comfortable and be supported in their choice. Midwives assisting women in pregnancy and childbirth should get trained to inform the pregnant women about FSP alternatives during pregnancy and encourage them to find a position that they find comfortable during pregnancy, labour, and birth. Health care providers and stakeholders in the health system are obligated to facilitate an enabling environment for the provision of respectful maternity care, including enabling various birth positions. A protocol for applying FSPs should be developed, as it is a new approach in Ethiopia that appears to provide quality maternal health care services that deliver improved maternal and neonatal outcomes. The regional health office should prepare a training package for the maternal health care providers to learn elements of new practice infrastructures, and materials needed should be fulfilled for the implementation of the new approach.

Maternal health care workers should assume that it is acceptable to allow women to adopt comfortable position when they deliver, and that may also offer protection from adverse maternal and neonatal outcomes. All clinicians working in maternity services have a responsibility to counsel pregnant mothers at every ANC visit and support laboring mothers to be allowed to choose a preferred birth position. This should be considered as one component of ANC services. Researchers should conduct qualitative studies from a maternal perspective. Future studies should have a reasonably long follow-up period starting from pregnancy with education about different birth positions to the postpartum period.

## Figures and Tables

**Table 1:** Comparison of the socio-demographic and economic characteristics of the study participants between the intervention and control group in Amhara public Health facilities, 2019.

Variables		Control Group (*N* = 360)	Intervention Group (*N* = 688)
Age	15–24 years	125 (34.7%)	240 (34.9%)
	25–34 years	190 (52.8%)	361 (52.5%)
	35–54 years	45 (12.5%)	87 (12.6%)
Residence	Urban Rural	257 (71.4%) 103 (28.6%)	473 (68.8%) 215 (31.3%)
Type of health facility	Hospital	280 (77.8%)	542 (78.8%)
	Health center	80 (22.2%)	146 (21.2%)
Marital status	Married	347 (96.4%)	648 (94.2%)
	Single	13 (3.6%)	40 (5.8%)
Religion	Orthodox	331 (91.9%)	621 (90.3%)
	Muslim	29 (8.1%)	67 (9.7%)
Educational status	Unable to read and write	108 (30.0%)	200 (29.1%)
	Able to read and write	40 (28.8%)	99 (71.2%)
	Grade 1–8	81 (36.3%)	142 (63.7%)
	Grade 9–12	88 (31.9%)	188 (68.1%)
	Diploma and above	43 (42.2%)	59 (57.8%)
Occupation	Housewife	230 (63.9%)	463 (67.3%)
	Daily laborer	22 (6.1%)	47 (6.8%)
	Private	68 (18.9%)	93 (13.5%)
	Government employee	40 (11.1%)	85 (12.4%)
Wealth Index	Very poor	66 (18.3%)	144 (20.9%)
	Poor	75 (20.8%)	134 (19.5%)
	Middle	72 (20%)	137 (19.9%)
	Rich	77 (21.4%)	134 (19.5%)
	Very rich	70 (19.4%)	139 (19.9%)

Chi-square test; with *p* < 0.05, No variable showed significant difference between the groups.

**Table 2:** Obstetric characteristics of the study participants in the intervention compared to control group, in Amhara public Health facilities, 2019.

Variables		Control Group (*N* = 360)	Intervention Group (*N* = 688)
Gravidity	1	162 (45.0%)	312 (45.3%)
	2–4	162 (45.0%)	300 (43.6%)
	≥5	36 (10%)	76 (11.1%)
Parity	1	167 (46.4%)	334 (48.5%)
	≥2	193 (53.6%)	354 (51.5%)
Number ANC follow-up	1–3	122 (37.8%)	263 (41.2%)
	4	201 (62.2%)	375 (58.8%)
BMI of mothers	<18.4 kg/m^2^	8 (2.2%)	37 (5.4%)
	18.5–24.99 kg/m^2^	281 (78.1%)	515 (74.9%)
	25–29.99 kg/m^2^	63 (17.5%)	116 (16.9%)
	>30 kg/m^2^	8 (2.2%)	20 (2.9%)

Chi-square test; with *p* < 0.05: No variable showed significant difference between the groups.

**Table 3:** Bi-variable Log-binomial regression analysis of effects of the intervention on the risk of perineal tear and Low APGAR score in Amhara Public Health facilities, 2019.

		Intervention Group *N* = 378 (%)	Control Group *N* = 592 (%)	RR (95%, CI)
Perineal tear	Yes	45 (11.9%)	124 (20.9%)	0.57 (0.41, 0.78) *
	No	333 (88.1%)	468 (79.1%)	1
APGAR score	<7 (Low APGAR score)	60 (15.6%)	168 (28.3%)	0.61 (0.42, 0.89) *
	7 and above	318 (84.4%)	424 (71.7%)	1

* stands for *p*-value < 0.05.

## Data Availability

The data set supporting the findings of this study are available in the form of tables and figures in the manuscript file. In case of further information needed it could be obtained from the corresponding author upon reasonable request.
